# Springhalt

**Published:** 1892-01

**Authors:** Williamson Bryden


### SPRINGHALT.*

WILLIAMSON BRYDEN, V. S.

About the 1st of June, 1891, I received a letter from Dr. Howard, president of the Massachusetts Veterinary Association, inviting me, with other veterinarians, to call at the wharf of the N. Ward Company and see a remarkable case of springhalt. It was intended to kill the subject within a few days and send the spinal cord to Worcester to be examined at the Clark University there. The invitation was accepted and the case carefully examined.

He was a small, black pony, about fourteen hands high and twenty years old, apparently well enough with the exception of his hind legs, which were badly affected with springhalt, both legs being nearly, if not quite, alike. In viewing him from behind, the *crests* of the *illium* were about even, the *gluteals* both alike flat, the *hips* drooping and the *sacrum* and tail elevated and coarse; *the muscles superior to the stiffle* were somewhat wasted; and *muscles behind the tibia* were small, and felt like tendons to within two or three inches of their origin; the *hocks were* both coarse, but not more than ought to be expected under the circumstances; his age, and the way he had been used for a long time, having tended to produce changes; both had a small elevation, or slight coarseness, at

* Report of a case at the Massachusetts Veterinary Association, November 25, 1891.

the seat of bone spavin. The legs afterwards became swollen below the hock, from the fact that the stall in which he stood part of the time, although at least five feet wide, was not wide enough to enable him to walk into it, and when once in, if he attempted to move, which was very frequently, he struck the outside of his ankles with such force against the sides of the stall that they became swollen, bruised and sore.

The parts that attracted my attention most, however, were his hoofs. They had evidently been causing the creature trouble for a long time, and were both alike, or nearly so. They were smaller than nature intended them to be, and nearly round, the wall short and very thick, the sole low, the bars strong, the heels short, and the frog of medium size. The horn seemed fairly healthy, but it grasped the extremity like a vice, causing him such uneasiness that often during the night he would dance about for several minutes at a time, making it difficult to enter the stall or go near him. Indeed, his attempts at locomotion were ludicrous, and he threw himself down several times, and screamed either from pain, fright, or irritation, due probably to some form of neuritis or neurosis.

I wrote Dr. Howard, through Dr. Austin Peters, the Secretary of the Massachusetts Veterinary Association, stating that I had seen the animal, and that having treated such cases, though never one so bad, I could demonstrate that the primary trouble was not in the spinal cord, which had very little seriously wrong with it, but in the feet. I also offered to take one leg, any one else who had a theory of his own or others, on the subject, taking the other. Nobody wanted it, however, so the case was kindly turned over to me, and on June 9th, 1891, I commenced treatment as follows :

A horse-shoer, with a sharp knife and rasp, was secured. The foot, when lifted, was held with great difficulty. The wall was thinned with the rasp all it would bear as high as the coronet. The sole was next levelled ; the inside wall, both from the fact that he toed out and his stamping his foot with great violence on the inner half was low. The soles were carefully trimmed, the commissures pared out, the bars thinned, and the heels opened. This greatly relieved the crowded structures within, and removed the mechanical pressure and interference with the blood vessels and nerves of the extremities. He was then left in charge of Mr. Andrew Ward, who took great interest in the case, furnishing keep and faithful attendants, who had orders to keep his feet continually in poultices. For two or three days he tolerated this treatment, and improved at least 50 per cent., but from his own efforts to get the poultices off, or from the bags being tied too tight, his pasterns became badly corded and so sore that he became almost unmanageable. Nothing further could be accomplished until these sores were healed. This was followed by a slight blister of bin-iodide of mercury ointment, applied above the coronet, which took three weeks to heal, during which time he was worse, if possible, than at the beginning. The blister was a mistake.

I then had him stand in mud during the day, and at night ordered his feet to be wrapped in long bandages soaked in lime water, linseed oil and soft potash soap, to which was added a little carbolic acid. These were neatly put on the hoofs in figure eight fashion, and kept soaked with the preparation. He tolerated this treatment and began to improve very rapidly, and on the day he was destroyed, July 27, from two to three weeks after I had stopped treatment, he was able to trot off after two or three twitches on starting, a quite natural gait, both on a straight course and in turning, something he was unable to do before being treated.

So far as I am aware, this discovery was made by my father over thirty years ago in Northern Vermont, where our family settled on arriving in this country from Scotland, and where we remained for six years, before removing to Boston in 1861. He was led to it by observing the large number of colts every spring with defective hoofs and legs, which he attributed mainly to the restraints and idleness incident to domestication, and the want of *tear and wear* to the hoofs during the long winter months, especially while the animals were growing. Many have treated the hoofs for defects and diseases of the feet, but no one before had ever grasped the grand conception that they dominated and determined the character of the limbs, and influenced the conformation of the body too. On my graduation from the Montreal Veterinary College, in 1871, I took up, at his request, the study of the subject, and submitted to the VETERINARIAN at least two short articles on springhalt, and other diseases peculiar to horses' limbs, and which I maintained were caused by defective conditions of their hoofs—the first in 1873, I think, the second in July, 1875. Since then I have contributed to about all of the veterinary journals published in Great Britian and America articles relating to animal locomotion and veterinary orthopedics, among which springhalt was always included.

There are several modifications of springhalt. This case presented features somewhat similar to what are sometimes seen in connection with bad forms of scratches, although when I first saw him, and up to the time when the poultice strings hurt him and he was placed in the narrow stall, there was no abrasion or evidence of soreness or inflamation of the skin. In other respects it showed all the symptoms of ordinary chronic springhalt.

In previous articles on this subject I have pointed out that the successful treatment of this disease depends on whether it first presents itself in *a very young animal* or in one that has reached *maturity*. The young animal, affected with it from early youth, if allowed to grow up without any effort to arrest or control the defect, will be found the most difficult to treat successfully after maturity, as inharmonies in the different parts of the leg are not easily regulated ; whereas, springhalt occuring at maturity or after, if not the result of a permanent injury, is very often quite as amenable to treatment as other erratic or equivocal gaits, such as paddling, interfering, over-reaching, hitching, pacing, etc., defects attributable to a similar class of circumstances and surroundings, and composing a family of diseases that have no relationship whatever to chorea, or other diseases originating in the brain or spinal cord.

Whatever part the hoof crowds in or interferes with, will react on some part of the limb above, causing changes, it may be in one or more muscles or parts, so that they act out of harmony. It may disturb a nerve, and destroy or modify adversely its influence, changing the gait or causing lameness. It may cause shortening of such parts as the flexor tendons and ligaments, and the animal walks on his toe or on the side of the foot. It may induce shortening of the metatarsal flexor, and cause it to perform more than its share of work, when bone spavin will follow. It may cause shortening and atrophy of the muscles of the hip, and the tail will almost invariably be carried to the shrunken side, instead of the side on which the muscles are strongest.

All those diseases of the limbs *recognized as peculiar to the horse have their origin there.* There we find the predisposing factor. The acquired defective hoof determines the character of the defect in the limb. General Grant, in his letter to Dunbar, endorsed this significance of the influence exerted by the hoof.

A springhalt gait from any other cause, is not springhalt. To class it with chorea, or to imagine it due to changes in the bones of the hock, is nonsense. The changes in the hock and elsewhere are coincident ; results, not causes.

Let members of the veterinary profession take up this great study of veterinary orthopedies, animal locomotion and organization, pedospedics, as Mr. Hubbell has named it, or by whatever name we finally decide to call the study, instead of all rushing into specialties in bacteriology, etc., and depend upon it, a domain containing treasures of such richness and such interest will be found that, if known more perfectly now, might have made the recent grand achievements of Sunol, Palo Alto, Allerton and Nelson several seconds faster. This is the science that is not only to save our phenomenal two-year-olds from decrepitude at five, but that is to make it possible for them to rival their early achievements when three times five.

Among our great horsemen a few have seemed to appreciate the importance of this glorious subject, more have tried to explain it, but none with the comprehensiveness to which it is entitled. The veterinary profession still has an opportunity to demonstrate the richness of some of the gems it contains. It ought to be one of the most important "chairs" in our veterinary educational institutions, and one that many among our influential people would delight in assisting to endow. Let us do original work here, and not look to France and Germany for anything new. Let us begin now, before someone has to teach us, and again carry off the honors.

